# 

**Published:** 1911-02

**Authors:** 


### CONTENTS.

ORIGINAL COMMUNICATIONS—

The Transfusion of Blood. Report of Twenty Cases. By H. P. Cole, M. D., Mobile, Ala. .... 551

Chronic Appendicitis with Report of Cases. W. L. Cook, M. D., Columbus, Ga. .... 556

Varicocele in the Male. By Aime Paul Heineck, M. D., Chicago, Ill. .. 560

Report of Case of Cardiospasm. By C. A. Dexter, M. D., Columbus, Ga. .... 572

A Clinical Review of Some Cases in Private Practice. By Thos. E. Mitchell, M. D., Columbus, Ga. .... 573

The Pathogenesis of Tabes Dorsalis. By Dr. T. A. Williams, Washington, D. C. .... 576

EDITORIALS .... 590

NEWS AND NOTES .... 591

BOOK REVIEWS .... 602

